# Food Composition Databases (FCDBs): A Bibliometric Analysis

**DOI:** 10.3390/nu15163548

**Published:** 2023-08-11

**Authors:** Andy Wai Kan Yeung

**Affiliations:** Oral and Maxillofacial Radiology, Applied Oral Sciences and Community Dental Care, Faculty of Dentistry, The University of Hong Kong, Hong Kong SAR, China; ndyeung@hku.hk

**Keywords:** food composition database, FCDB, bibliometric, VOSviewer, flavonoid

## Abstract

Food composition databases (FCDBs) are important tools that provide information on the nutritional content of foods. Previously, it was largely unclear what nutritional contents and which FCDBs were involved in highly cited papers. The bibliometric study aimed to identify the most productive authors, institutions, and journals. The chemicals/chemical compounds with high averaged citations and FCDBs used by highly cited papers were identified. In July 2023, the online database Web of Science Core Collection (WoSCC) was queried to identify papers related to FCDBs. A total of 803 papers were identified and analyzed. The first paper indexed in WoSCC was published in 1992 by Pennington, which described the usefulness of FCDB for researchers to identify core foods for their own studies. In that paper, the FCDB described was the USDA 1987–88 NFCS (the United States Department of Agriculture 1987–88 Nationwide Food Consumption Survey). The most productive author was Dr. Paul M. Finglas, the Head of the Food Databanks National Capability at the Quadram Institute (Norwich, UK) and the Managing Director of EuroFIR. His most cited paper among this dataset was about the development of an online Irish food composition database together with EuroFIR. The most productive institutions were the USDA and the World Health Organization (WHO) instead of universities. Flavonoid was the most recurring chemical class among the highly cited ones. The anti-oxidative properties and protective effects against heart disease and cancer of flavonoids might be some of the reasons for their popularity in research. Among the highly cited papers, the most heavily used FCDBs were the USDA database for the flavonoid content of selected foods, Fineli, the USDA National Nutrient Database for Standard Reference (USNDB), EuroFIR eBASIS-Bioactive Substances in Food Information Systems, and Phenol-Explorer. High-quality national and international FCDBs should be promoted and made more accessible to the research and public communities to promote better nutrition and public health on a global scale.

## 1. Introduction

Food composition databases (FCDBs) are important tools that provide information on the nutritional content of foods (mainly simple and non-cooked processed foods), including macronutrients (e.g., carbohydrates, proteins, and fats), micronutrients (e.g., vitamins and minerals), and other components (e.g., dietary fiber and water) [1,2]. Nutritional assessment via diet analysis requires two steps: evaluating food consumption qualitatively and quantitatively, followed by converting the food into nutrient intake with the aid of FCDBs [3].

Besides nutritional content, the non-nutrient components have also gained attention in recent years, some of which are called bioactive compounds that exist in plant-based food items and have “health promoting/beneficial and/or toxic effects when ingested” [4]. The data stored in FCDBs is used by nutritionists, dietitians, and researchers to assess the nutritional quality of diets, plan meals, and evaluate how food intake is associated with health [5]. Food manufacturers can also utilize FCDBs to develop and label products [6], and policymakers can use them to derive dietary guidelines and regulations [7].

It was believed that the very first food composition table was published back in 1818 by Percy and Vaquelin [8], according to Church [9]. Since then, many countries have developed their own databases, and there are now several global databases available. One very prominent online tool was developed by the European Food Information Resource Network of Excellence (EuroFIR) called FoodExplorer [10], which allows access to multiple national FCDBs mainly based in Europe but also in North America, South Africa, Australia, and Japan.

At first glance, it would seem intuitive to think that FCDBs should have international/global coverage so that all researchers and users could refer to identical reference values compiled from a single, standardized dataset. However, there are several challenges. For instance, there is a lack of standardization in food analysis methods [11]. Different laboratories may adopt different analysis methods for food samples, resulting in variations in the reported nutrient content. Moreover, some foods may not be commonly consumed or available around the food laboratories involved in the sampling, resulting in a lack of data or limited data. These scenarios could lead to data inconsistency and incompleteness, rendering it difficult for a FCDB to cover all food items on a global scale. On the other hand, national FCDBs can be very useful, as they can be compiled to record the details of major and even minor food items and food dishes consumed on a national level [12,13,14]. Besides, there exists regional variability in food composition due to many factors, such as climate, technologies, soil, and different cultivars, and hence using a national or regional FCDB could sometimes be more accurate [11].

Global harmonization in methodology is certainly needed among regional databases to allow data interchange. One important initiative in Europe was the formation of the EuroFIR Association Internationale Sans But Lucratif (AISBL, meaning an international non-profit association), which had a mission to “promote harmonization and exploitation of high-quality food composition data and foster cooperation and participation in development with national compiler organizations” [15]. Formed in 2009, EuroFIR AISBL advocates for improved data quality, storage, and accessibility for food information in Europe and the rest of the world. A prior bibliometric analysis showed that over 100 papers published since 2005 mentioned EuroFIR, and they were mainly published in journals dealing with Agricultural and Biological Sciences, Nursing, Medicine, and Chemistry [15].

With the growing literature dealing with FCDBs, it would be beneficial to examine the relevant literature from a bibliometric perspective so that the most productive researchers and institutions could be identified for further knowledge exchange and research collaboration. The highly cited chemicals/chemical compounds could be identified to reveal what nutritional contents received more attention by the scientific community. Moreover, the exact FCDBs used by the highly cited papers were identified, so that future studies and follow-up studies could choose the same FCDBs for easier comparison or choose different FCDBs with different coverage of food items and parameters, depending on their research aims.

## 2. Materials and Methods

In July 2023, the online database Web of Science Core Collection (WoSCC) was accessed with the following search string: FCDB OR FCDBs OR “food composition datab*” WoS is a comprehensive literature database with a long history and is the most widely used database in bibliometrics [16]. The search string was applied to the title, abstract, and author keyword fields of indexed publications. No additional filters were placed on other bibliographic aspects, such as publication year or publication language (Table 1). The search yielded 803 publications.

Publication and citation counts were extracted directly from the WoSCC database. Counties from England, Scotland, North Ireland, and Wales were merged to represent the United Kingdom. The complete records of the publications were exported into VOSviewer (version 1.6.19, Centre for Science and Technology Studies of Leiden University, Leiden, The Netherlands) [17] for processing and visualization of a term map, with default parameters applied. In brief, the “Create a map based on text data” function was chosen. Then the option “Read data from bibliographic database files” was chosen. In the “Choose fields” step, title and abstract fields were chosen, and the options “Ignore structured abstract labels” and “Ignore copyright statements” were checked. Binary counting of the terms was chosen. The term map showed the recurring terms from the title and abstract of the analyzed publications. To improve visual clarity, the map showed terms appearing in at least 1% (*n* = 8) of the publications only, a threshold commonly adopted by previous studies [18,19,20]. Each term is labeled a node, with the node size indicating the publication count, its color indicating the citations per publication (CPP), and the inter-node distance indicating their frequency of co-occurrence.

## 3. Results

**Figure 1:**
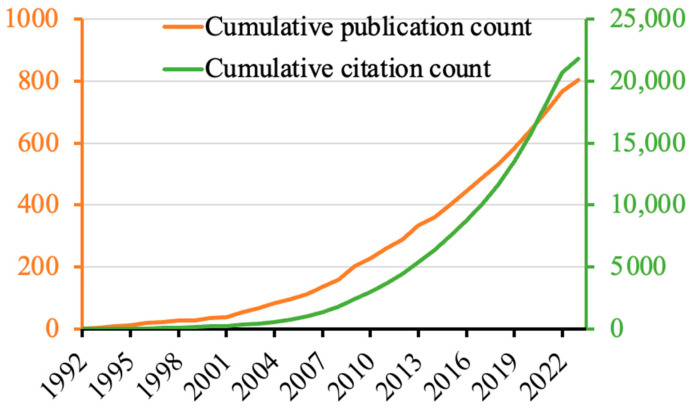
Cumulative publication and citation count of FCDB research.

The cumulative publication and citation counts of the FCDBs research are shown in Figure 1. The 803 papers were cited 21,813 times in total, with an h-index of 76 and a CPP of 27.2). The first paper indexed in WoSCC was published in 1992 by Pennington [21] and described the usefulness of FCDB for researchers to identify core foods for their own studies. In that paper, the FCDB described was the USDA 1987–88 NFCS (the United States Department of Agriculture 1987–88 Nationwide Food Consumption Survey). Since this paper was published, the publication and citation counts have gradually increased over the years. The ratio of original articles (*n* = 677, CPP = 28.4) to reviews (*n* = 56, CPP = 42.2) was 12:1. The top 5 most productive authors, institutions, countries, journals, and journal categories are listed in Table 2. The most productive author was Dr. Paul M. Finglas, the Head of the Food Databanks National Capability at the Quadram Institute (Norwich, UK) and the Managing Director of EuroFIR. His most cited paper among this dataset was about the development of an online Irish food composition database together with EuroFIR [22]. Interestingly, the most productive institutions were the USDA and the World Health Organization (WHO) instead of universities. Meanwhile, the most productive countries were led by the United States, followed by European countries and Australia. The top five most productive journals consisted of some traditional journals, such as the Journal of Food Composition and Analysis (started in 1987; 2022 impact factor = 4.3; Q2 in JCR Food Science and Technology and Chemistry, Applied), as well as newer journals, such as Nutrients (started in 2009; 2022 impact factor = 5.9; Q1 in JCR Nutrition and Dietetics).

**Figure 2:**
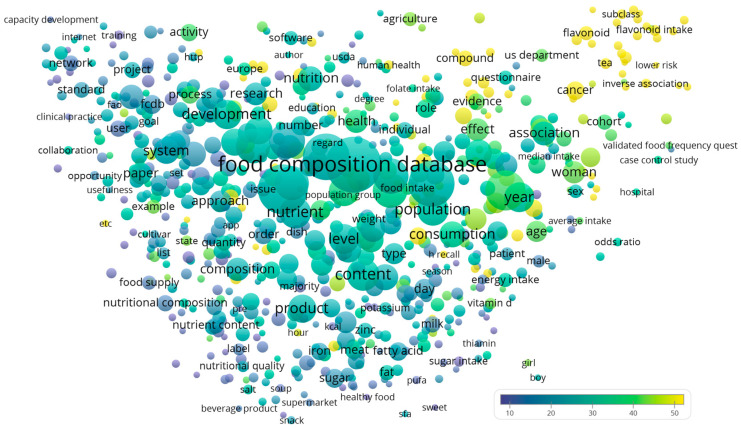
Term map showing recurring terms from titles and abstracts of FCDB publications.

The recurring terms from the title and abstract of the analyzed publications are visualized as a term map in Figure 2. Terms with the highest CPP (yellow nodes) were concentrated at the top right corner of the figure. Many of these terms were chemicals or compounds (Table 3).

The FCDBs used as the data source or involved in the methodology among the top 50 most cited papers were recorded (Table 4). The top five FCDBs were the USDA database for the flavonoid content of selected foods, Fineli, the USDA National Nutrient Database for Standard Reference (USNDB), EuroFIR eBASIS-Bioactive Substances in Food Information Systems, and Phenol-Explorer.

The top 10 most-cited papers are reported here. Many of them concerned flavonoids and polyphenols and ranged from establishing new FCDBs to using data from existing FCDBs for their analysis (Table 5).

## 4. Discussion

The bibliometric analysis of FCDBs literature has found that the 803 papers were cited 21,813 times in total, with an h-index of 76 and a CPP of 27.2. The ratio of original articles to reviews was 12:1. The most productive institutions were USDA and WHO instead of universities. This was largely different from related research fields such as nutraceuticals and functional foods [56] and ethnopharmacology [57], both of which were dominated by university research. Among the top 50 most cited papers, the heavily used FCDBs were the USDA database for the flavonoid content of selected foods, Fineli, the USDA National Nutrient Database for Standard Reference (USNDB), EuroFIR eBASIS-Bioactive Substances in Food Information Systems, and Phenol-Explorer.

FCDBs are important resources for multiple stakeholders that provide detailed information on the nutritional composition of food items, such as data on essential nutrients, vitamins, minerals, and other bioactive components present in the diverse food products indexed by the databases. The information contained in FCDBs could be applied in numerous fields, including nutrition, healthcare, food science, agriculture, and public health.

As the top five recurring journal categories were Nutrition Dietetics, Food Science Technology, Chemistry Applied, Public Environmental Occupational Health, and Endocrinology Metabolism, the relevance of FCDBs to some of these research fields would be briefly covered here. In nutrition and dietetics, dieticians could rely on FCDBs to develop personalized meal plans and provide dietary recommendations for individuals with specific dietary needs. In food science, FCDBs also provide valuable information on the nutritional composition of raw materials and food dishes. One Indonesian study devised a “low sodium, high potassium” healthy diet based on information from the Indonesian food composition database; however, found that a high potassium and high fiber diet made the menu more expensive [58]. Moreover, the food industry could rely on FCDBs for product development and marketing, whereas consumers could make informed food choices and have increased trust in the validity of food product nutritional labels. The Food Label Information Program (FLIP) from the University of Toronto was a good example [6]. It provided comprehensive food product nutrition information (from package labels) for Canadian pre-packaged food and beverages. In terms of public health, FCDBs could play an important role in the development and assessment of public health policies and interventions. For instance, a large-scale study in France referred the dietary records of participants to a FCDB to check the extent of processing of the food they consumed and found that the intake of ultra-processed foods was associated with a gain in body mass index and a higher risk of overweight as well as obesity [59]. As such, the government might promote the consumption of minimally processed foods. Besides, the data provided by FCDBs could assist governments and international agencies in devising food fortification programs and designing strategies to address malnutrition. Nutritional data on edible insects, for instance, were entered into the FAO/INFOODS Food Composition Database for Biodiversity (BioFoodComp) so that people could have a reliable data source regarding the protein and micronutrient contents of the common species [60]. For endocrinology and metabolism, FCDBs could provide data in epidemiological research to elucidate the associations between diet, nutrition, and health outcomes by estimating nutrient intake and associating it with the incidence of chronic diseases, such as cardiovascular diseases and type 2 diabetes. One pan-European study demonstrated an inverse association between flavonoids, especially flavanols and flavonols, and the incidence of type 2 diabetes [61]. It suggested the beneficial role of flavonoids in preventing diabetes.

The information from FCDBs could also be relevant to agriculture. Usually, the nutritional values of vegetables listed in FCDBs do not consider or reflect seasonal variations. For instance, one study identified vitamin C rich vegetables from the USNDB and sampled them in different seasons [62]. Results found that vitamin C was much higher in winter-sampled spinach, potatoes in summer/fall, and oranges in winter/spring, implying that the average values stored in FCDBs might be over- or under-estimated when seasonal changes are considered. Accurate knowledge of the nutritional composition of crops is essential for agricultural planning and decision-making. FCDBs help in identifying nutrient-rich crop varieties and promoting their cultivation to enhance food security and combat malnutrition. Additionally, they provide insights into the potential impact of climate change and agricultural practices on food composition, allowing for the development of adaptive strategies to safeguard the nutritional quality of crops.

## 5. Limitations

This bibliometric study had some limitations. First, this study relied on a single database, WoSCC. This was conducted because each database records citation counts differently. However, publications not indexed by WoSCC would be missed. Second, by means of “obliteration by incorporation” [63,64], some older papers would not be cited by newer papers anymore when the initial findings have been regarded as “common sense” or “general knowledge” by the current standard. Hence, the citation count might not completely reflect the impact of some papers. Meanwhile, VOSviewer also had its limitations, such as the inability to pre-define some word phrases to be counted or to identify the exact papers that contributed to the counts of a particular term or country.

## 6. Conclusions

Overall, FCDBs are very important resources and tools for stakeholders in many fields, such as nutrition, food science, public health, and healthcare. FCDBs supported laboratory and human research studies, evidence-based policymaking, and consumer education. Many of the FCDB papers dealt with flavonoids, whose anti-oxidative properties and protective effects against heart disease and cancer might be some of the reasons for their popularity in research. Meanwhile, one of the most commonly used FCDBs among the most cited papers was the USDA database for the flavonoid content of selected foods. High-quality national and international FCDBs should be promoted and made more accessible to the research and public communities to promote better nutrition and public health on a global scale.

## Figures and Tables

**Table 1:** Search strategy in the Web of Science.

Databases searched	Science Citation Index Expanded (1970–present) Social Sciences Citation Index (1956–present) Arts and Humanities Citation Index (1975–present) Conference Proceedings Citation Index–Science (2009–present) Conference Proceedings Citation Index–Social Science and Humanities (2009–present) Emerging Sources Citation Index (2005–present)
Search string	FCDB OR FCDBs OR “food composition datab*” (* is a truncation symbol)
Fields searched	Title, abstract, and author keywords
Timespan	All years (1956–2023)

**Table 2:** The top five most productive authors, institutions, countries, journals, and journal categories.

	Number of Publications (% of 803)	Citations per Publication (CPP)
Author		
Finglas, Paul M	19 (2.4)	20.7
Amiano, Pilar	16 (2.0)	62.4
Zamora-Ros, Raul	16 (2.0)	61.7
Charrondiere, U. Ruth	15 (1.9)	44.1
Khaw, Kay-Tee	15 (1.9)	56.7
Trichopoulou, Antonia	15 (1.9)	62.4
Institution		
United States Department of Agriculture (USDA)	39 (4.9)	50.3
World Health Organization	37 (4.6)	36.6
Netherlands National Institute for Public Health and the Environment	35 (4.4)	34.8
UK Research Innovation (UKRI)	32 (4.0)	34.2
University of Sydney	32 (4.0)	21.7
Country/region		
United States	151 (44.4)	44.4
United Kingdom	110 (35.6)	35.6
Italy	94 (42.1)	42.1
Australia	85 (16.7)	16.7
Spain	85 (32.7)	32.7
Journal		
Journal of Food Composition and Analysis	152 (18.9)	21.0
Nutrients	66 (8.2)	15.3
Food Chemistry	59 (7.3)	28.2
British Journal of Nutrition	38 (4.7)	48.1
European Journal of Clinical Nutrition	28 (3.5)	41.6
Journal category		
Nutrition Dietetics	437 (54.4)	27.6
Food Science Technology	312 (38.9)	26.5
Chemistry Applied	222 (27.6)	24.8
Public Environmental Occupational Health	47 (5.9)	27.0
Endocrinology Metabolism	35 (4.4)	24.1

**Table 3:** Notable highly cited chemicals or compounds in the analyzed publications.

Chemicals or Compounds	Number of Publications (% of 803)	Citations per Publication (CPP)
Flavonoid	37 (4.6)	107.4
Beta carotene	26 (3.2)	57.4
Carotenoid	21 (2.6)	69.9
Flavonol	17 (2.1)	106.8
Flavan	16 (2.0)	72.9
Isoflavone	16 (2.0)	60.9
Flavone	15 (1.9)	92.3
Lignan	15 (1.9)	72.7
Lycopene	15 (1.9)	68.2
Proanthocyanidin	15 (1.9)	140.7
Polyphenol	14 (1.7)	114.4
Tocopherol	14 (1.7)	100.1
Anthocyanin	13 (1.6)	103.8
Flavanone	13 (1.6)	132.5
Alpha carotene	10 (1.2)	66.5
Beta cryptoxanthin	10 (1.2)	74.1
Anthocyanidin	9 (1.1)	95.4
Flavanol	8 (1.0)	101.5
Vitamin k	8 (1.0)	66.6

**Table 4:** Food composition databases (FCDBs) are used by the top 50 most cited papers. It should be noted that some databases had multiple updates/versions.

FCDB	Website or Citation to the FCDB	Number of Publications
USDA database for the flavonoid content of selected foods	[23]	7
Fineli	[24]	6
USDA National Nutrient Database for Standard Reference (USNDB)	[25]	6
EuroFIR eBASIS-Bioactive Substances in Food Information Systems	[26]	4
Phenol-Explorer	[27]	4
Ciqual	[28]	2
Singapore Food Composition Database	[29]	2
USDA-Iowa State University database on the isoflavone content of foods	[30]	2
Australian food composition database (AUSNUT2007)	[31]	1
DAta Food Networking-DAFNE database (Not available online anymore)	[32]	1
Database for the glucosinolate content of cruciferous vegetables	[33]	1
Database of levels of nitrosamines, heterocyclic amines, and polycyclic aromatic hydrocarbons in foods	[34]	1
Database of resveratrol and piceid in Spanish foods	[35]	1
EFSA Comprehensive European Food Consumption Database	[36]	1
FAO/INFOODS Food Composition Database for Biodiversity	[37]	1
FLAVIOLA Flavanol Food Composition database	[38]	1
Harvard University Food Composition Database	[39]	1
Italian Food Composition Database	[40]	1
Korean food composition database	[41]	1
LEBTAB (Germany)	No reference or link provided	1
PhytoHub	[42]	1
Rice antioxidants database	[43]	1
UK database for the phylloquinone (vitamin K1) content of foods	[44]	1
USDA Database for the Proanthocyanidin Content of Selected Foods	[45]	1
USDA Dietary Source Nutrient Database	[46]	1
Vegetal Estrogens in Nutrition and the Skeleton (VENUS) (Not available online anymore)	[47]	1

**Table 5:** Summary of the top 10 most-cited papers.

Paper	Food Compound	Paper Type	Summary	Number of Citations
[48]	Flavonoids	Review	It was an overview of dietary flavonoids, covering the nomenclature, occurrence, and intake, and reviewed the estimated intakes of selected subclasses of flavonoids in several countries based on data from FCDBs. This paper commented that teas provide rich dietary sources of flavan-3-ols, flavonols, and derived tannins in many countries, but FCDB values for tannin derivatives are “weak at best and in most cases nonexistent”.	590
[49]	Flavonols, flavones, and flavanones	Review	It covered the evidence from epidemiological studies on the association between human health and the intake of flavonols, flavones, and flavanones, such as a risk reduction of age-related chronic diseases ranging from cancer to cardiovascular disease and other chronic conditions. The authors concluded that clinically controlled trials should be conducted to further test the associations identified from these epidemiological studies.	374
[50]	Polyphenols	Review	It briefed readers on the chemistry, occurrence, and human health of polyphenols. It covered the most common classification of the phenolic compounds into two major groups: flavonoids and non-flavonoids called polyphenols. Due to their applications in food preservation and therapeutic usage, much research has been conducted to elucidate the association between their intake and numerous diseases, ranging from diabetes, hypertension, cardiovascular disease, and cancer.	371
[43]	Rice antioxidants	Article	By compiling data from over 300 papers, this article built a FCDB on the contents of various antioxidants contained in rice, such as phenolic acids, flavonoids, gamma-oryzanol, anthocyanins, proanthocyanidins, phytic acid, tocopherols, and tocotrienols. It also highlighted that black rice had the highest antioxidant activities, followed by purple, red, and brown varieties, and japonica varieties had a higher antioxidant content than indica varieties. The FCDB was constructed based on data/values provided by published papers around the world, so the presence of certain food compounds and values depended on their availability in the existing literature.	365
[29]	Non-specific	Article	It described the development, validation, and calibration of a quantitative food frequency questionnaire designed to target Singapore Chinese and the subsequent development of a FCDB for analyzing the collected dietary data.	330
[51]	Tocopherol, tocotrienol, and plant sterol	Article	It reported the tocopherol, tocotrienol, and plant sterol contents of 14 vegetable and 9 industrial fats/oils commercially available in Finland. Results were compared to the values listed by Fineli and the USDA National Nutrient Database.	308
[52]	Polyphenols	Article	It collected dietary records from adults to estimate the quantity of dietary intake and the major sources of polyphenols in Finland. Results found that phenolic acids comprised the dominant group of polyphenol intake, followed by proanthocyanidins, anthocyanidins, and other flavonoids. Coffee, cereals, berries, and fruits were the major sources.	302
[53]	Phytosterol	Article	It analyzed the phytosterol composition of nuts and seeds commonly available in the United States. Results found that sesame seed and wheat germ had the highest phytosterol content, whereas Brazil nuts had the lowest. Among the common snack foods, pistachios, and sunflower kernels had the highest phytosterol levels, though they were behind sesame seed and wheat germ.	284
[54]	Non-specific	Review	It was before the establishment of EuroFIR, and the authors suggested that the development of a pan-European FCDB should be considered to standardize the quantification method, the determination of the consumption pattern of individual foods, and the integration of the likelihoods of large amounts of consumption and chemical quantity at these high levels.	296
[55]	Berry phenolics	Review	It was a review of the antioxidant and antimicrobial activities of berry phenolics in Finland. Pieces of evidence from studies of cranberries, cultivated and wild blueberries, black currants, cloudberries, lingonberries, and red raspberries were discussed.	261

## Data Availability

The data used for the analysis in this study is available from the Web of Science online database upon subscription.

## References

[1] Marconi S., Durazzo A., Camilli E., Lisciani S., Gabrielli P., Aguzzi A., Gambelli L., Lucarini M., Marletta L. (2018). Food composition databases: Considerations about complex food matrices. Foods.

[2] Delgado A., Issaoui M., Vieira M.C., Saraiva de Carvalho I., Fardet A. (2021). Food composition databases: Does it matter to human health?. Nutrients.

[3] Merchant A.T., Dehghan M. (2006). Food composition database development for between country comparisons. Nutr. J..

[4] Gry J., Black L., Eriksen F.D., Pilegaard K., Plumb J., Rhodes M., Sheehan D., Kiely M., Kroon P.A. (2007). EuroFIR-BASIS—A combined composition and biological activity database for bioactive compounds in plant-based foods. Trends Food Sci. Technol..

[5] Ene-Obong H., Schönfeldt H.C., Campaore E., Kimani A., Mwaisaka R., Vincent A., El Ati J., Kouebou P., Presser K., Finglas P. (2019). Importance and use of reliable food composition data generation by nutrition/dietetic professionals towards solving Africa’s nutrition problem: Constraints and the role of FAO/INFOODS/AFROFOODS and other stakeholders in future initiatives. Proc. Nutr. Soc..

[6] Ahmed M., Schermel A., Lee J., Weippert M., Franco-Arellano B., L’Abbé M. (2022). Development of the food label information program: A comprehensive Canadian branded food composition database. Front. Nutr..

[7] Gurinovic M., Milesevic J., Zekovic M., Kadvan A., Ranic M., Glibetic M. (2020). Capacity development in food and nutrition in central and Eastern Europe: A decade of achievements. Food Policy.

[8] Percy P., Vacquelin N. (1818). Sur la qualité nutritive des aliments comparés entre eux. Bull. Fac. Med. Paris.

[9] Church S. (2006). The history of food composition databases. Nutr. Bull..

[10] Finglas P.M., Berry R., Astley S. (2014). Assessing and improving the quality of food composition databases for nutrition and health applications in Europe: The contribution of EuroFIR. Adv. Nutr..

[11] Greenfield H., Southgate D.A. (2003). Food Composition Data: Production, Management, and Use.

[12] Durazzo A., Camilli E., Marconi S., Lisciani S., Gabrielli P., Gambelli L., Aguzzi A., Lucarini M., Kiefer J., Marletta L. (2019). Nutritional composition and dietary intake of composite dishes traditionally consumed in Italy. J. Food Compost. Anal..

[13] Durazzo A., Lisciani S., Camilli E., Gabrielli P., Marconi S., Gambelli L., Aguzzi A., Lucarini M., Maiani G., Casale G. (2017). Nutritional composition and antioxidant properties of traditional Italian dishes. Food Chem..

[14] Durazzo A., Lucarini M., Santini A., Camilli E., Gabrielli P., Marconi S., Lisciani S., Aguzzi A., Gambelli L., Novellino E. (2019). Antioxidant properties of four commonly consumed popular Italian dishes. Molecules.

[15] Durazzo A., Astley S., Kapsokefalou M., Costa H.S., Mantur-Vierendeel A., Pijls L., Bucchini L., Glibetić M., Presser K., Finglas P. (2022). Food composition data and tools online and their use in research and policy: EuroFIR AISBL contribution in 2022. Nutrients.

[16] Zhu J., Liu W. (2020). A tale of two databases: The use of Web of Science and Scopus in academic papers. Scientometrics.

[17] van Eck N.J., Waltman L. (2010). Software survey: VOSviewer, a computer program for bibliometric mapping. Scientometrics.

[18] Yeung A.W.K. (2023). The Revived Interest in Ageusia Research during the COVID-19 Pandemic: A Bibliometric Analysis. Life.

[19] Yeung A.W.K., Tosevska A., Klager E., Eibensteiner F., Tsagkaris C., Parvanov E.D., Nawaz F.A., Völkl-Kernstock S., Schaden E., Kletecka-Pulker M. (2022). Medical and health-related misinformation on social media: Bibliometric study of the scientific literature. J. Med. Internet Res..

[20] Yeung A.W.K., Parvanov E.D., Nawaz F.A., Rayan R.A., Kletecka-Pulker M., Willschke H., Atanasov A.G. (2022). COVID-19 Rapid Antigen Tests: Bibliometric Analysis of the Scientific Literature. Int. J. Environ. Res. Public Health.

[21] Pennington J.A. (1992). Total diet studies: The identification of core foods in the United States food supply. Food Addit. Contam..

[22] Black L., Ireland J., Møller A., Roe M., Walton J., Flynn A., Finglas P., Kiely M. (2011). Development of an on-line Irish food composition database for nutrients. J. Food Compost. Anal..

[23] USDA Database for the Flavonoid Content of Selected Foods. https://data.nal.usda.gov/dataset/usda-database-flavo-noid-content-selected-foods-release-32-november-2015.

[24] Fineli. https://fineli.fi/fineli/en/index.

[25] USDA National Nutrient Database for Standard Reference (USNDB). https://data.nal.usda.gov/dataset/usda-national-nutrient-database-standard-reference-legacy-release.

[26] EuroFIR eBASIS-Bioactive Substances in Food Information Systems. https://ebasis.eurofir.org/Default.asp.

[27] Phenol-Explorer. http://phenol-explorer.eu/.

[28] Ciqual. https://ciqual.anses.fr/.

[29] Hankin J.H., Stram D.O., Arakawa K., Park S., Low S.-H., Lee H.-P., Yu M.C. (2001). Singapore Chinese Health Study: Development, validation, and calibration of the quantitative food frequency questionnaire. Nutr. Cancer.

[30] USDA-Iowa State University Database on the Isoflavone Content of Foods. https://data.nal.usda.gov/dataset/usda-database-isoflavone-content-select-ed-foods-release-21-november-2015.

[31] Australian Food Composition Database (AUSNUT2007). https://www.foodstandards.gov.au/science/monitoringnutrients/ausnut/Pages/ausnut2007.aspx.

[32] Data Food Networking-DAFNE Database. http://www.nut.uoa.gr/.

[33] McNaughton S., Marks G. (2003). Development of a food composition database for the estimation of dietary intakes of glucosinolates, the biologically active constituents of cruciferous vegetables. Br. J. Nutr..

[34] Database of Levels of Nitrosamines, Heterocyclic Amines, and Polycyclic Aromatic Hydrocarbons in Foods. http://epic-spain.com/libro.html.

[35] Zamora-Ros R., Andres-Lacueva C., Lamuela-Raventós R.M., Berenguer T., Jakszyn P., Martínez C., Sanchez M.J., Navarro C., Chirlaque M.D., Tormo M.-J. (2008). Concentrations of resveratrol and derivatives in foods and estimation of dietary intake in a Spanish population: European Prospective Investigation into Cancer and Nutrition (EPIC)-Spain cohort. Br. J. Nutr..

[36] EFSA Comprehensive European Food Consumption Database. https://www.efsa.europa.eu/en/data-report/food-consumption-data#the-efsa-comprehen-sive-european-food-consumption-database.

[37] FAO/INFOODS Food Composition Database for Biodiversity. https://www.fao.org/infoods/infoods/tables-and-databases/faoinfoods-databases/en/.

[38] Vogiatzoglou A., Mulligan A.A., Luben R.N., Lentjes M.A., Heiss C., Kelm M., Merx M.W., Spencer J.P., Schroeter H., Kuhnle G.G. (2014). Assessment of the dietary intake of total flavan-3-ols, monomeric flavan-3-ols, proanthocyanidins and theaflavins in the European Union. Br. J. Nutr..

[39] Harvard University Food Composition Database. https://www.hsph.harvard.edu/nutrition-questionnaire-service-center/.

[40] Italian Food Composition Database. http://www.bda-ieo.it/wordpress/en/.

[41] Korean Food Composition Database. http://koreanfood.rda.go.kr/eng/fctFoodSrchEng/engMain.

[42] PhytoHub. https://phytohub.eu/.

[43] Goufo P., Trindade H. (2014). Rice antioxidants: Phenolic acids, flavonoids, anthocyanins, proanthocyanidins, tocopherols, tocotrienols, γ-oryzanol, and phytic acid. Food Sci. Nutr..

[44] Bolton-Smith C., Price R.J., Fenton S.T., Harrington D.J., Shearer M.J. (2000). Compilation of a provisional UK database for the phylloquinone (vitamin K1) content of foods. Br. J. Nutr..

[45] USDA Database for the Proanthocyanidin Content of Selected Foods. https://data.nal.usda.gov/data-set/usda-database-proanthocya-nidin-content-selected-foods-release-2-2015.

[46] O’Neil C.E., Keast D.R., Fulgoni III V.L., Nicklas T.A. (2012). Food sources of energy and nutrients among adults in the US: NHANES 2003–2006. Nutrients.

[47] Vegetal Estrogens in Nutrition and the Skeleton (VENUS). https://www.venus-ca.org.

[48] Beecher G.R. (2003). Overview of dietary flavonoids: Nomenclature, occurrence and intake. J. Nutr..

[49] Graf B.A., Milbury P.E., Blumberg J.B. (2005). Flavonols, flavones, flavanones, and human health: Epidemiological evidence. J. Med. Food.

[50] Durazzo A., Lucarini M., Souto E.B., Cicala C., Caiazzo E., Izzo A.A., Novellino E., Santini A. (2019). Polyphenols: A concise overview on the chemistry, occurrence, and human health. Phytother. Res..

[51] Schwartz H., Ollilainen V., Piironen V., Lampi A.-M. (2008). Tocopherol, tocotrienol and plant sterol contents of vegetable oils and industrial fats. J. Food Compost. Anal..

[52] Ovaskainen M.-L., Torronen R., Koponen J.M., Sinkko H., Hellstrom J., Reinivuo H., Mattila P. (2008). Dietary intake and major food sources of polyphenols in Finnish adults. J. Nutr..

[53] Phillips K.M., Ruggio D.M., Ashraf-Khorassani M. (2005). Phytosterol composition of nuts and seeds commonly consumed in the United States. J. Agric. Food Chem..

[54] Kroes R., Müller D., Lambe J., Löwik M., Van Klaveren J., Kleiner J., Massey R., Mayer S., Urieta I., Verger P. (2002). Assessment of intake from the diet. Food Chem. Toxicol..

[55] Heinonen M. (2007). Antioxidant activity and antimicrobial effect of berry phenolics—A Finnish perspective. Mol. Nutr. Food Res..

[56] Yeung A.W.K., Mocan A., Atanasov A.G. (2018). Let food be thy medicine and medicine be thy food: A bibliometric analysis of the most cited papers focusing on nutraceuticals and functional foods. Food Chem..

[57] Yeung A.W.K., Heinrich M., Atanasov A.G. (2018). Ethnopharmacology—A bibliometric analysis of a field of research meandering between medicine and food science?. Front. Pharmacol..

[58] Farapti F., Buanasita A., Atmaka D.R., Setyaningtyas S.W., Adriani M., Rejeki P.S., Yamaoka Y., Miftahussurur M. (2022). Potassium intake is associated with nutritional quality and actual diet cost: A study at formulating a low sodium high potassium (LSHP) healthy diet. J. Nutr. Sci..

[59] Beslay M., Srour B., Méjean C., Allès B., Fiolet T., Debras C., Chazelas E., Deschasaux M., Wendeu-Foyet M.G., Hercberg S. (2020). Ultra-processed food intake in association with BMI change and risk of overweight and obesity: A prospective analysis of the French NutriNet-Santé cohort. PLoS Med..

[60] Nowak V., Persijn D., Rittenschober D., Charrondiere U.R. (2016). Review of food composition data for edible insects. Food Chem..

[61] Zamora-Ros R., Forouhi N.G., Sharp S.J., Gonzalez C.A., Buijsse B., Guevara M., van der Schouw Y.T., Amiano P., Boeing H., Bredsdorff L. (2013). The association between dietary flavonoid and lignan intakes and incident type 2 diabetes in European populations: The EPIC-InterAct study. Diabetes Care.

[62] Phillips K.M., Tarrago-Trani M.T., McGinty R.C., Rasor A.S., Haytowitz D.B., Pehrsson P.R. (2018). Seasonal variability of the vitamin C content of fresh fruits and vegetables in a local retail market. J. Sci. Food Agric..

[63] Merton R.K. (1965). On the Shoulders of Giants: A Shandean Postscript.

[64] Yeung A.W.K. (2021). Is the influence of Freud declining in psychology and psychiatry? A bibliometric analysis. Front. Psychol..

